# Suprapatellar Versus Infrapatellar Approach for Intramedullary Nailing of Tibial Shaft Fractures: A Comparative Analysis of Clinical and Functional Outcomes

**DOI:** 10.7759/cureus.96823

**Published:** 2025-11-14

**Authors:** Giovanni Longo, Andrea Modesti, Andrea Vespasiani, Domenico Topa

**Affiliations:** 1 Department of Orthopedics and Traumatology, Sapienza University of Rome, Rome, ITA; 2 Department of Life, Health and Environmental Sciences, University of L'Aquila, L'Aquila, ITA; 3 Department of Orthopedics and Traumatology, San Giovanni Addolorata Hospital, Rome, ITA

**Keywords:** anterior knee pain, diaphyseal tibia fracture, infrapatellar nailing, lysholm score, suprapatellar nailing

## Abstract

Background

With the increasing adoption of the suprapatellar approach for intramedullary tibial nailing in diaphyseal tibial fractures, this study compared intraoperative and postoperative outcomes between infrapatellar nailing (IPN) and suprapatellar nailing (SPN).

Methods

A retrospective analysis was conducted on 51 SPN and 58 IPN procedures performed at a single trauma center between October 2018 and June 2023. Operative time, postoperative blood loss, functional outcomes using the Lysholm score, and the incidence of anterior knee pain (AKP) at follow-up were compared.

Results

There were no significant differences in age or sex between the groups. Mean follow-up was 36.74 ± 18.11 months for SPN and 35.98 ± 15.11 months for IPN. Mean operative time was significantly shorter in the SPN group (79.7 ± 34.0 min) compared to the IPN group (91.9 ± 36.0 min, p < 0.05). The SPN group showed significantly higher Lysholm scores (89.45 ± 9.2 vs. 85.1 ± 10.5, p = 0.017) and a lower incidence of AKP. No significant difference was observed in postoperative blood loss.

Conclusion

Compared to the IPN approach, SPN for diaphyseal tibial fractures may reduce operative time, decrease the incidence of AKP, and improve postoperative functional outcomes.

## Introduction

Diaphyseal tibial fractures are relatively common injuries that can result from high-energy trauma in young patients or low-energy trauma in the elderly [1]. Intramedullary nailing is the most frequently used technique for the treatment of these fractures and represents the standard of care for diaphyseal tibial fractures, offering satisfactory clinical and functional recovery [2]. Plate osteosynthesis is a valid alternative, mainly indicated when the articular surface is involved in distal tibial fractures [3]. The traditional approach for intramedullary nailing is the infrapatellar (IP) approach, either through a patellar tendon split or by retracting the tendon medially or laterally [4]. Proximal third tibial diaphyseal fractures can be challenging to treat using the IP approach because adequate entry point visualization requires hyperflexion of the knee, which can lead to further fracture displacement [4]. Additionally, anterior knee pain (AKP) is a known complication of the IP approach, affecting between 10% and 40% of patients [5].

The suprapatellar (SP) approach was developed to overcome these difficulties. The semi-extended knee position allows for proper limb support without the need for continuous manual fracture reduction, simplifies intraoperative fluoroscopic imaging, and overall results in a more efficient procedure without compromising outcomes [6,7]. The SP approach is therefore associated with lower postoperative AKP, shorter operative times [6], and better postoperative functional outcomes as measured by the Lysholm score [8-17]. Moreover, SP tibial nailing appears to be associated with a lower total radiation dose (TRD) compared to the IP approach [9].

This study was designed to compare outcomes between SP and IP tibial nailing. The aim was to evaluate whether suprapatellar nailing (SPN) was associated with shorter operative time, reduced incidence of AKP, improved functional outcomes, and less postoperative blood loss compared to infrapatellar nailing (IPN). We, therefore, assessed operative duration, incidence of AKP, postoperative blood loss, and functional outcomes using the Lysholm score [8].

## Materials and methods

This study included 109 consecutive adult patients (≥18 years old) who underwent intramedullary nailing for diaphyseal tibial fractures at San Giovanni Addolorata Hospital in Rome between October 2018 and June 2023. All patients with acute diaphyseal fractures of the tibia treated with intramedullary nailing were eligible for inclusion. Only diaphyseal tibial fractures were included to ensure homogeneity, as proximal and distal fractures have distinct anatomical and biomechanical characteristics and are associated with higher malalignment risk when treated with intramedullary nailing [18-20]. Exclusion criteria comprised polytrauma, the need for concomitant plastic surgery, previous surgical procedures involving the tibia, pre-existing tibial deformities, metastatic bone disease, and a history of tibial nonunion. A total of 51 patients underwent SPN and were compared to 58 patients treated with IPN. All surgeries were performed by three experienced orthopedic trauma surgeons using standardized operative protocols for both SP and IP approaches. Fracture types were classified according to the Arbeitsgemeinschaft für Osteosynthesefragen (AO) classification [21] for diaphyseal tibial fractures, using imaging studies and clinical records. Patient demographics were collected from both paper and electronic medical records. All procedures were performed at a single trauma center. The implants used were the DePuy Synthes Expert Tibial Nail (DePuy Synthes, Warsaw, IN, USA). In SPN cases, the DePuy Synthes SP instrumentation system for the Expert Tibial Nail was used. All patients received perioperative antibiotic prophylaxis.

Patients were positioned supine on a radiolucent operating table. For the IP approach, a lateral support and leg holder were used; for the SP approach, a foam wedge was used to maintain the knee in semi-extension. Knee hyperflexion is not always necessary during IPN. When provisional reduction is maintained with Poller screws, clamps, or temporary K-wires, guidewire placement can be achieved with reduced flexion angles, minimizing deforming forces and maintaining reduction accuracy [22,23]. Operative time, including fracture reduction, was recorded for both techniques. Postoperative blood loss was calculated based on the difference between preoperative and 48-hour postoperative hemoglobin levels. Postoperative rehabilitation followed a uniform institutional protocol including early range-of-motion exercises and progressive weight-bearing as tolerated.

Postoperatively, patients were contacted and asked to complete the Lysholm Knee Scoring Scale [8]. All clinical evaluations and Lysholm score assessments were performed by the same trained orthopedic resident under the supervision of the senior authors to minimize inter-observer variability. The Lysholm Knee Scoring Scale is a validated, freely available instrument that does not require prior permission for academic use. It was applied without modification and cited according to its original publication. AKP was assessed at rest and during kneeling. Patients were also asked about limping, joint locking, instability, and difficulty or pain when climbing stairs or squatting.

Statistical analyses were performed using IBM SPSS Statistics for Windows, Version 26 (Released 2018; IBM Corp., Armonk, New York, United States)​​​​​​. A p-value < 0.05 was considered statistically significant.

## Results

A total of 109 surgical procedures were identified during the selected time period. An additional six cases were excluded due to meeting one or more of the exclusion criteria. The SPN group included 51 patients with a mean age of 50 years (range: 19-91), of whom 13 were female (approximately 25%). The IPN group included 58 patients with a mean age of 49 years (range: 18-89), of whom 16 were female (approximately 27%).

All patients underwent intramedullary nailing (IMN) for acute tibial fracture; no cases of nonunion were included. The mean follow-up duration was 36.74 ± 18.11 months in the SPN group and 35.98 ± 15.11 months in the IPN group. Using the t-test and chi-square analysis, no significant differences were observed between the SPN and IPN groups in terms of age, sex, or fracture type (open vs. closed) (Table 1).

**Table 1:** Patient demographics and injury characteristics by group.

Variable	Infrapatellar Group	Suprapatellar Group	P-value
Median age (years)	49 (18-89)	50 (19-91)	0.77
Male:female ratio	42:16	38:13	0.97
Closed:open fractures	44:14	48:13	0.87

Fractures were classified according to the AO classification [21], with the distribution presented in Table 2.

**Table 2:** Fracture classification according to AO/OTA system. SPN: suprapatellar nailing; IPN: infrapatellar nailing; AO/OTA: Arbeitsgemeinschaft für Osteosynthesefragen/Orthopaedic Trauma Association

Fracture pattern	Simple 42-A	Wedge 42-B	Complex 42-C
SPN	26	15	10
IPN	30	16	12

The most common injury mechanisms were falls from height (52%, n=56) and sports-related trauma (25%, n=27). Most patients were classified as ASA grade I or II (85%).

**Figure 1:**
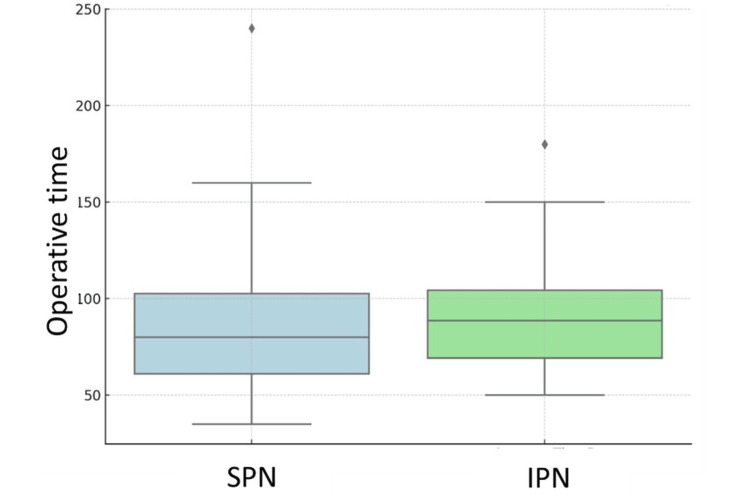
Comparison of operative time between suprapatellar nailing (SPN) and infrapatellar nailing (IPN) approaches.

The mean operative time was significantly shorter in the SPN group compared to the IPN group (79.7 ± 34.0 minutes vs. 91.9 ± 36.0 minutes, respectively; p < 0.05) (Table 3 and Figure 1).

**Table 3:** Operative time. SPN: suprapatellar nailing; IPN: infrapatellar nailing

	IPN	SPN	P-value
Operative time (minutes)	91.9 ± 36.0	79.7 ± 34.0	<0.05

The mean difference in hemoglobin levels (pre- vs. post-operative) was -1.53 g/dL (range: -4.20 to -0.70) in the SPN group and -1.27 g/dL (range: -3.50 to -0.40) in the IPN group. No statistically significant difference was observed between the two groups using both the independent samples t-test (p = 0.158) and the Mann-Whitney U test (p = 0.148).

**Figure 2:**
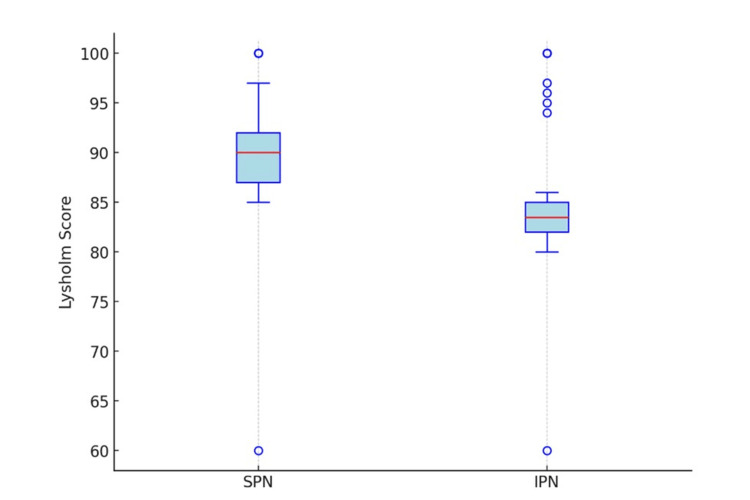
Comparison of Lysholm scores between patients treated with suprapatellar nailing (SPN) and infrapatellar nailing (IPN).

**Figure 3:**
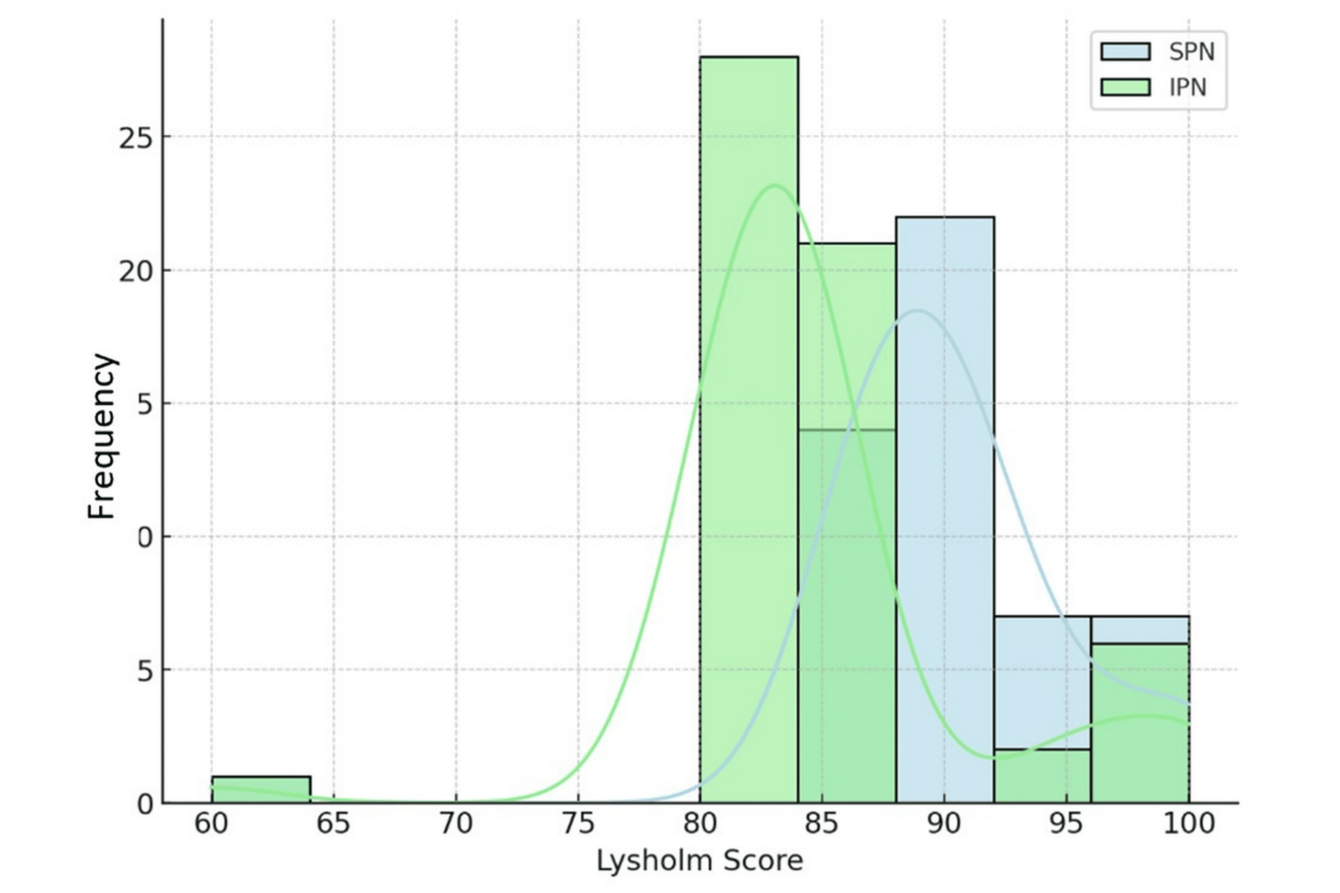
Distribution of Lysholm scores - suprapatellar nailing (SPN) versus infrapatellar nailing (IPN) with density curves.

At outpatient follow-up, all patients were asked to complete the Lysholm knee scoring questionnaire [8], focusing on AKP and return to baseline function. An independent samples t-test revealed significantly higher postoperative Lysholm scores in the SPN group (mean 89.45 ± 9.2) compared to the IPN group (mean 85.1 ± 10.5; p = 0.017) (Figures 2-3).

Median scores were higher in the SPN group, which also showed lower dispersion among the lower values. Although some outliers were observed, with scores ranging between 60 and 65, the overall trend favored the SPN group.

The SPN group demonstrated a higher concentration of scores within the 90-100 range, while the IPN group showed a broader distribution that included lower scores. At follow-up, AKP was reported in four patients in the SPN group and in 14 patients in the IPN group, representing a statistically significant difference (p = 0.037) and indicating a lower incidence of AKP in the SPN group.

## Discussion

Surgical treatment of distal tibial fractures can be performed using various techniques, including external fixation, plating, and intramedullary nailing. IMN has become increasingly favored for distal tibia fractures due to its minimally invasive nature, reduced soft tissue disruption, lower rates of malunion, and superior biomechanical stability [24].

This study focused exclusively on diaphyseal tibial fractures, consistent with prior literature comparing SP and IP approaches, in which intramedullary nailing represents the standard of care [18-20]. Compared with previous studies, which typically enrolled between 40 and 90 patients and often included mixed fracture patterns, our cohort of 109 patients with strictly diaphyseal fractures provides one of the largest homogeneous samples available, enhancing the reliability of comparative outcomes between the SP and IP approaches [6,11-13].

Our findings support the clinical efficacy of the SP approach for tibial IMN compared to the IP approach, particularly in terms of operative time, incidence of AKP, and short-term functional outcomes. The significantly reduced operative duration observed in the SPN group (79.7 ± 34.0 minutes vs. 91.9 ± 36.0 minutes; p < 0.05) is consistent with previous literature [6,7,9], suggesting that the semi-extended limb position facilitates improved intraoperative control and easier fluoroscopic imaging.

A particularly noteworthy finding was the significantly lower incidence of AKP in the SPN group (7.8%) versus the IPN group (24.1%) (p = 0.037), as also reported by other authors [25]. However, given that the SP approach involves transquadriceps entry and subpatellar passage, postoperative pain and technical difficulty may increase in patients with pre-existing osteoarthritis [26].

Multiple factors may contribute to AKP, including nail prominence, incision site, Hoffa’s fat pad, injury to the IP branch of the saphenous nerve, and intra-articular damage [15-17,27,28]. The SP approach, being anatomically distant from the IP incision site, may help reduce AKP incidence.

Concerns remain regarding the risk of iatrogenic intra-articular injury with the SPN technique [16,17,28]. However, some authors suggest that this risk is minimal when a protective sleeve is used during nail insertion [29]. Eastman et al. [30] argued that joint pressure from the SP approach does not cause chondrocyte necrosis. Supporting this, Sanders et al. [25] performed arthroscopy in 15 patients who had undergone SPN; 13 (86.7%) showed normal cartilage appearance, and only two patients had grade II cartilage lesions. Follow-up MRI at one year revealed resolution of these lesions. These findings support the biomechanical rationale of the SP technique, which avoids manipulation of the patellar tendon and reduces the risk of anterior joint irritation or direct injury [5,10,13].

Although no statistically significant difference was observed in postoperative blood loss (measured by hemoglobin variation), the SPN group showed a trend toward lower mean blood loss (-1.53 g/dL vs. -1.27 g/dL), suggesting a potential advantage that warrants further investigation with larger sample sizes.

Lastly, functional outcomes as measured by the Lysholm score [8] were significantly better in the SPN group, both in terms of mean score (89.45 ± 9.2 vs. 85.1 ± 10.5; p = 0.017) and overall score distribution. These data, coupled with reduced AKP, suggest faster and more satisfactory recovery in the short term for patients treated with the SP approach, even in complex fracture patterns. Based on these results, the SP technique should be considered the preferred option, especially in proximal third diaphyseal fractures or in patients at higher risk for AKP. However, further randomized prospective studies with long-term follow-up are needed to confirm these findings and to assess potential chondral damage in the patellofemoral joint. This study has some limitations that should be acknowledged. First, it is a retrospective analysis conducted at a single center, which may limit the generalizability of the findings to other settings. In addition, the absence of randomization may introduce potential selection bias; however, all procedures were performed by experienced surgeons using a standardized institutional protocol to minimize variability. Although the follow-up period was sufficient to assess mid-term clinical outcomes, it does not allow definitive conclusions regarding late complications or long-term evolution. Nevertheless, we believe that the present results provide a valuable contribution to the existing literature, and future prospective, multicenter studies with longer follow-up are warranted to confirm and expand these observations.

## Conclusions

The SP approach for intramedullary nailing of tibial shaft fractures demonstrated significant clinical benefits compared to the traditional IP technique. In this retrospective series, SPN was associated with shorter operative times, improved postoperative functional outcomes as measured by Lysholm scores, and a significantly lower incidence of AKP, without an increase in postoperative blood loss. These findings support the use of the SP approach as a safe and effective alternative, particularly in proximal diaphyseal fractures or in patients at higher risk of AKP. Further prospective, randomized studies with larger cohorts and longer follow-up are warranted to confirm these results and to better evaluate potential effects on intra-articular structures.
